# Associations between depression and different measures of obesity (BMI, WC, WHtR, WHR)

**DOI:** 10.1186/1471-244X-13-223

**Published:** 2013-09-12

**Authors:** Jörg Wiltink, Matthias Michal, Philipp S Wild, Isabella Zwiener, Maria Blettner, Thomas Münzel, Andreas Schulz, Yvonne Kirschner, Manfred E Beutel

**Affiliations:** 1 Department of Psychosomatic Medicine and Psychotherapy, University Medical Center of the Johannes Gutenberg-University, Mainz, Germany; 2 Center for Thrombosis and Hemostasis, University Medical Center of the Johannes Gutenberg-University, Mainz, Germany; 3 Department of Medicine 2, University Medical Center of the Johannes Gutenberg-University, Mainz, Germany; 4 Institute of Medical Biostatistics, Epidemiology and Informatics, University Medical Center of the Johannes Gutenberg-University, Mainz, Germany

**Keywords:** Obesity, Abdominal obesity, Anthropometric measures, Depression, Somatic-affective symptoms

## Abstract

**Background:**

Growing evidence suggests that abdominal obesity is a more important risk factor for the prognosis of cardiovascular and metabolic diseases than BMI. Somatic-affective symptoms of depression have also been linked to cardiovascular risk. The relationship between obesity and depression, however, has remained contradictory. Our aim was therefore to relate body mass index (BMI) and different measures for abdominal obesity (waist circumference, WC, waist-to-hip ratio, WHR, waist-to-height ratio, WHtR) to somatic vs. cognitive-affective symptoms of depression.

**Methods:**

In a cross-sectional population based study, data on the first N = 5000 participants enrolled in the Gutenberg Health Study (GHS) are reported. To analyze the relationship between depression and obesity, we computed linear regression models with the anthropometric measure (BMI, WC, WHR, WHtR) as the dependent variable and life style factors, cardiovascular risk factors and psychotropic medications as potential confounders of obesity/depression.

**Results:**

We found that only the somatic, but not the cognitive-affective symptoms of depression are consistently positively associated with anthropometric measures of obesity.

**Conclusions:**

We could demonstrate that the somatic-affective symptoms of depression rather than the cognitive-affective symptoms are strongly related to anthropometric measures. This is also true for younger obese starting at the age of 35 years. Our results are in line with previous studies indicating that visceral adipose tissue plays a key role in the relationship between obesity, depression and cardiovascular disease.

## Background

Although relations between obesity and somatic conditions are unambiguous [1,2], there is an ongoing debate about the usefulness of the Body Mass Index (BMI) as a prognostic factor for cardiovascular and metabolic disease. While, BMI can be measured easily and reliably (only weight and height measures are necessary), there are some concerns about the reliability of the measurement of waist or hip circumference depending on the precise site at which they are measured [3]. BMI was a strong predictor for overall mortality in a sample of 900.000 participants of 57 prospective studies from different countries (92% of the participants were in Europe, Israel, the USA, or Australia). Each gain of 5 kg/m^2^ was associated with a 40% increase of vascular mortality [4].

Growing evidence of comparison studies, however, suggests that abdominal obesity is a more important risk factor for cardiovascular and metabolic diseases than general obesity. A recent prospective study with a total of 6.355 participants compared BMI, waist circumference (WC), waist-to-height ratio (WHtR) and waist-to-hip ratio (WHR); WHtR was the best predictor of cardiovascular risk and mortality (followed by WC and WHR) [5]. Based on the finding that the measure of abdominal fat provided a better assessment of risk of death among people with lower BMI (in a study with 360.000 participants), screening of WC and WHR was suggested in addition to BMI [6].

Visceral adipose tissue seems to play a key role in cardiovascular diseases (CVD), obesity and depression. As reviewed by Shelton & Miller, however, the underlying inflammatory mechanisms are complex and not fully understood [7] (for an overview). Studies have shown that visceral fat produces a higher amount of cytokines than subcutaneous fat [8]. High levels of cytokines such as TNF-alpha, IL-6 and C reactive protein have been found both in visceral obesity and in depression [9]; this finding has been replicated consistently [7]. An unhealthy life style, diabetes, CVD and inflammation may induce vascular damage in the brain, which may also lead to depressive symptoms [10]. Another potential mechanism linking obesity and depression is a dysregulation of the hypothalamic-pituitary-adrenal axis e.g. [11].

The relations between obesity and comorbid mental conditions are still discussed controversially. An increased prevalence of depression and other mental disorders in obese was found in several studies e.g. [12-14] and confirmed by a meta-analysis including seventeen community based studies with more than 200.000 participants [15]; the causal link between depression and obesity is supposed to be bidirectional [16]. Others, however, could not find an increased prevalence for mental disorders in the obese at all. e.g. [17-19]. However, only few studies have compared different anthropometric measures, even though they are quantitative, non-invasive and easy to determine techniques for determining body fat composition.

The abdominal fat distribution (measured by WHR) appeared to be the key mediator of the relationship between obesity and depression in a large cross sectional study with 65.648 adults [20]. A recent report of a large community sample of older (70 to 79 years old) participants could demonstrate the increased risk of onset of depressive symptoms in initially non-depressed obese men, but not for obese women during a 5 year follow up. Men with high visceral fat had a more than 2-fold increased risk of being persistently depressed compared to those with a normal amount of visceral fat; the association with depressive symptoms was more consistently related to abdominal obesity than to overall obesity [21].

Recent studies have shown that somatic-affective (e.g. feeling tired or having little energy), but not cognitive-affective (e.g. feeling down, depressed, or hopeless) symptoms of depression predicted poor medical outcomes in patients with cardiovascular disease e.g. [22]. However, this distinction has not been made by the aforementioned studies [20,21]. In a Dutch study with 1284 participants (50 to 70 years old) a positive correlation was found between all different measures of depressive symptoms (Beck Depression Inventory) and BMI, although the association between measures of visceral obesity (WC and WHR) was primarily driven by the somatic-affective symptom cluster [23].

Contradictory findings regarding the relationships between obesity and depression may be due to (a) the different measures of obesity (BMI or abdominal fat distribution) used, (b) the different measures of depression, esp. somatic-affective vs. cognitive-affective symptoms of depression and (c) potential sex differences (d) the u-shaped relationship between body weight and depression (e.g. [23-25]).

Therefore, our aim was to compare BMI as a measure for general obesity with different anthropometric measures for abdominal obesity (WC, WHR, WHtR) to somatic-affective and cognitive-affective depressive symptoms in a cross-sectional population based sample. We expected a closer relationship to depression in the measures of abdominal obesity compared to BMI and for somatic-affective vs cognitive-affective symptoms of depression.

## Methods

### Procedure and study sample

We investigated cross-sectional data of the first N = 5000 participants enrolled in the Gutenberg Health Study (GHS) from April 2007 to October 2008. The GHS is a population-based, prospective, observational single-center cohort study in the Rhine-Main-Region in western Mid-Germany. The GHS has been approved by the local ethics committee and by the local and federal data safety commissioners. The primary aim of the study is to evaluate and improve cardiovascular risk stratification. The sample was drawn randomly from the local registry in the city of Mainz and the district of Mainz-Bingen. The sample was stratified 1:1 for gender and residence and in equal strata for decades of age. Inclusion criteria were age 35 to 74 years and written informed consent. Persons with insufficient knowledge of German language, or those who reported that they were not able to visit the study center on their own (due to their physical and/or mental condition) were excluded. The response rate (defined as the recruitment efficacy proportion, i.e. the number of persons with participation in or appointment for the baseline examination divided by the sum of number of persons with participation in or appointment for the baseline examination plus those with refusal and those who were not contactable) was 60.3%. Due to the ongoing recruitment of the GHS, which is conducted in waves, a final statement concerning the response rate cannot be made at this time. According to the protocol, descriptive analysis of the cross-sectional data was planned for the first 5000 participants of the study. The design and the rationale of the Gutenberg Health Study (GHS) have been described in detail elsewhere [26].

### Materials and assessment

The 5-hour baseline-examination in the study center comprised evaluation of prevalent classical cardiovascular risk factors and clinical variables, a computer-assisted personal interview, laboratory examinations from a venous blood sample, blood pressure and anthropometric measurements. In general, all examinations were taken out according to standard operating procedures (SOPs) by certified medical technical assistants.

### Primary outcome measures

#### Depression

Depression was measured by the Patient Health Questionnaire (PHQ-9); caseness was defined by a score ≥ 10 with a sensitivity of 81% and a specificity of 82% for depressive disorder [27]. The somatic-affective and cognitive-affective dimensions of depression were defined according to prior studies [28-30]. Four PHQ-9 items related to problems with sleep, fatigability, appetite, and psychomotor agitation/retardation were classified as somatic-affective symptoms, whereas 5 items, related to lack of interest, depressed mood, negative feelings about self, concentration problems and suicidal ideation, were classified as cognitive-affective symptoms of depression [31]. While we were aware, that dimensions of depression (cognitive-affective and somatic-affective) in the community might differ from those in cardiovascular settings, we used the same dimensions for comparison purposes and due to their high face validity and comparability.

#### Obesity (anthropometric measures)

Weight, height, waist and hip circumference were measured according to a written, standardized manual. Waist circumference (WC) was measured with a tape measure midway between the lowest rib and the pelvis in position of expiration, hip circumference was measured at the widest circumference of the hip. The different anthropometric measures were calculated: BMI (weight in kg divided by the square of height in meters); WC (in cm); waist-to-hip ratio (WHR): WC divided by hip circumference; waist-to-height ratio (WHtR): WC divided by measured height in cm.

### Potential confounders

In addition to age and sex analyses were adjusted for a wide range of potential confounders (e.g. [32]). We predefined a comprehensive set of confounders with a potential relation to obesity and/or depression. These confounders can be grouped into life style factors, classical cardiovascular risk factors and psychotropic medication.

#### Life style factors

*Physical activity* was inquired with the “Short Questionnaire to assess health-enhancing physical activity” (SQUASH) [33]. The SQUASH captures commuting, leisure time, household, work and school activities. The questionnaire does not measure energy expenditure, but indicates the habitual activity level. Sleeping, lying, sitting and standing were classified as inactivity [34].

*The socioeconomic status* (SES) was defined according to Lampert’s and Kroll’s Scores of SES range from 3 to 27 while 3 indicates the lowest SES and 27 the highest SES [35]. Additionally, participants reported, whether they were living in a *partnership*. *Alcohol use* with a regular daily consumption of 10 g or more for female participants and 20 g or more for male participants was defined as heavy [36].

#### Classical cardiovascular risk factors

Cardiovascular risk factors were defined as follows e.g. [21,23]: *Smoking* was dichotomized into non-smokers (never smoker and ex-smoker) and smokers (occasional smoker, i.e. <1 cigarette/day, and smoker, i.e. >1 cigarette/day). *Diabetes* was defined in individuals with a definite diagnosis of diabetes by a physician or a blood glucose level of ≥ 126 mg/dl in the baseline examination after an overnight fast of at least 8 hours or a blood glucose level of ≥ 200 mg/dl after a fasting period < 8 hours. *Dyslipidemia* was defined as a definite diagnosis of dyslipidemia by a physician or an LDL/HDL-ratio of >3.5. *Hypertension* was diagnosed, if antihypertensive drugs were taken, or a mean systolic blood pressure of ≥140 mmHg (diastolic blood pressure ≥ 90 mmHg) in the 2nd and 3rd standardized measurement after 8 and 11 minutes of rest. A positive family history of myocardial infarction (FH-MI) was defined as at least one myocardial infarction in a female first-degree relative of <65 years or a male first-degree relative of <60 years.

#### Psychotropic medication

The following *psychotropic medications* potentially affecting mood and/or body weight were chosen as confounders [37]: non-selective monoamine reuptake inhibitors, selective serotonin reuptake inhibitor, other antidepressants, antipsychotics, anxiolytics, hypnotics/sedatives, antiepileptics, opioids.

### Statistical analysis

Statistical analysis was done by SAS for Windows 9.2 TS Level 1M0 (SAS Institute Inc.) Cary, NC, USA. Data are presented as numbers/percentage, mean (and 1.96-fold standard deviation) or median (and 25/75th percentile) as appropriate.

To analyze the relationship between depression and obesity, we computed separate linear regression models with the anthropometric measure (BMI, WC, WHR, WHtR) as the dependent variable and life style factors, cardiovascular risk factors and psychotropic medications as potential confounders of obesity/depression. We adjusted for life style factors (SES, partnership, physical activity, alcohol use), the traditional cardiovascular risk factors (smoking status, diabetes, dyslipidemia, hypertension, family history of myocardial infarction) and psychopharmacological treatments. Depressive symptoms were evaluated with two separate analyses: a) including the depression sum score (PHQ) and b) including the somatic-affective and the cognitive-affective symptoms. All p-values correspond to 2-tailed tests. As this is an explorative study no adjustments were made for multiple comparisons. Due to the large number of tests applied in this study p-values have to be interpreted with caution and in connection with effect size estimates.

## Results

### Sample characteristics

Table 1 shows the sociodemographic characteristics (age, partnership, SES), depressive symptoms, cardiovascular risk factors (smoking status, physical activity, weight, height, waist circumference, obesity, hypertension, dyslipidemia, diabetes, and family history of MI), alcohol use and psychotropic medication. In order to analyze linear relations between depression and obesity and because of the known u-shaped association between depression and body weight [23-25], underweight participants (BMI < 18.5 kg/m^2^) were excluded from analyses (N = 30).

**Table 1:** **Sample characteristics **^1)^

	**Total N = 4907-4970 **^3)^
	**% (N)**
**Sex, female**	49.1 (2438)
**Age**, in years; mean (age-range 35–74)	55.5
**Partnership**, yes	82.4 (4093)
**SES,** mean (± 1.96 SD)	12.6 (3.9, 21.3)
**Depression** (PHQ; > = cut-off of 10)	7.2 (354)
Total Score, median (Q1, Q3)	3.0 (1.0, 6.0)
Somatic-affective symptoms, median (Q1, Q3)	2.0 (1.0, 3.0)
Cognitive-affective symptoms, median (Q1, Q3)	1.0 (0, 2.0)
**Smoking status,** current	19.2 (950)
Physical activity, mean (± 1.96 SD) ^2)^	7349.2 (−218.3, 14916.7)
Alcohol (heavy use)	28.4 (1407)
Somatic conditions	
Obesity (BMI > =30)	24.2 (1204)
BMI, mean (± 1.96 SD)	27.3 (17.9, 36.6)
Waist circumference (cm), mean (± 1.96 SD)	93.9 (67.0, 120.7)
WHtR, mean (± 1.96 SD)	0.55 (0.40, 0.71)
WHR, mean (± 1.96 SD)	0.93 (0.74, 1.11)
Hypertension	51.5 (2558)
Dyslipidemia	29.4 (1460)
Diabetes	7.5 (371)
Family history of myocardial infarction	17.8 (883)
Psychotropic medication	
Non-selective monoamine reuptake inhibitors	2.3 (113)
Selective serotonin reuptake inhibitor	1.7 (85)
Other antidepressants	1.1 (55)
Antipsychotics	0.7 (33)
Anxiolytics	1.1 (54)
Hypnotics/sedatives	1.3 (66)
Antiepileptics	1.2 (59)
Opioids	1.6 (80)

Presented are percent (%) and number (N) resp. mean and ± 1.96 standard deviation or median (1st, 3rd quartile); ^1)^ Participants with BMI < 18.5 were excluded (N = 30); ^2)^ N = 986 missing; ^3)^ missings in physical activity not included in total N.

The mean age of the participants was 55.5 years (range 35–74 years). 2532 were male (50.9%), and 2438 participants were female (49.1%). The majority of the participants reported a low level of education (less than 10th grade). About 1/3 had completed high school. 24.5% had a college or university degree. Due to our inclusion criteria (age ≥ 35) pension was common with 33.1%, only 2.2% of the participants were unemployed.

We computed linear regression models with the anthropometric measure as a continuous dependent variable. Separately for men and women total depression, somatic-affective and cognitive-affective symptoms of depression were used as predictors. The model was additionally adjusted by life style factors, cardiovascular risk factors and psychotropic medications. Table 2 gives an overview over the results.

**Table 2:** Linear regression analysis of depression and anthropometric parameters stratified for sex

	**Female (N = 1850-1853)**				**Male (N = 2073-2075)**				**Total (N = 3922-3928)**			
	**Beta**	**B**	**SE**	**p**	**Beta**	**B**	**SE**	**p**	**Beta**	**B**	**SE**	**p**
**BMI**												
Depression (PHQ)	0.066	0.0968	0.0345	**0.0050**	0.043	0.0564	0.0275	**0.0400**	0.055	0.0762	0.0219	**0.0005**
Somatic-affective symptoms	0.137	0.3583	0.0715	**<.0001**	0.094	0.2165	0.0584	**0.0002**	0.121	0.2980	0.0460	**<.0001**
Cognitive-affective symptoms	−0.067	−0.1786	0.0746	**0.0167**	−0.046	−0.1076	0.0595	0.0708	−0.061	−0.1538	0.0473	**0.0012**
**WC**												
Depression	0.064	0.2365	0.0857	**0.0058**	0.048	0.1777	0.0766	**0.0204**	0.049	0.1972	0.0571	**0.0006**
Somatic-affective symptoms	0.122	0.8012	0.1780	**<.0001**	0.124	0.8184	0.1624	**<.0001**	0.116	0.8289	0.1201	**<.0001**
Cognitive-affective symptoms	−0.054	−0.3632	0.1861	0.0511	−0.071	−0.4789	0.1655	**0.0039**	−0.062	−0.4603	0.1237	**0.0002**
**WHtR**												
Depression	0.064	0.0015	0.0005	**0.0043**	0.052	0.0011	0.0004	**0.0086**	0.056	0.0013	0.0003	**0.0001**
Somatic-affective symptoms	0.126	0.0053	0.0011	**<.0001**	0.104	0.0040	0.0009	**<.0001**	0.119	0.0048	0.0007	**<.0001**
Cognitive-affective symptoms	−0.057	−0.0025	0.0012	**0.0318**	−0.046	−0.0018	0.0009	0.0513	−0.057	−0.0024	0.0007	**0.0013**
**WHR**												
Depression	0.026	0.0005	0.0005	0.2720	0.069	0.0015	0.0004	**0.0008**	0.115	0.0009	0.0003	**0.0035**
Somatic-affective symptoms	0.053	0.0018	0.0010	0.0600	0.107	0.0040	0.0009	**<.0001**	0.060	0.0029	0.0007	**<.0001**
Cognitive-affective symptoms	−0.025	−0.0019	0.0010	0.3761	−0.029	−0.0011	0.0009	0.2322	−0.023	−0.0017	0.0007	0.0908

Models adjusted for age, socioeconomic status (SES), partnership, smoking status, physical activity, diabetes, dyslipidemia, hypertension, family history of myocardial infarction, alcohol (heavy use), psychotropic medication (non-selective monoamine reuptake inhibitors, selective serotonin reuptake inhibitor, other antidepressants, antipsychotics, anxiolytics, hypnotics/sedatives, antiepileptics, opioids), bold type p < .05.

Depression (PHQ-9 total score) was significantly related to all different measures of obesity, except for WHR in women. The somatic-affective symptoms of depression and all anthropometric measures - again except for WHR in women - were consistently related. These relations were stronger than those with the total score. The cognitive-affective symptoms of depression were negatively related or unrelated to the anthropometric measures. All standardized regression coefficients (beta) of somatic-affective depressive symptoms were larger than those for the corresponding cognitive-affective symptoms. Table 2 gives an overview.

## Discussion and conclusions

The aim of our study was to compare BMI as a measure for general obesity with different anthropometric measures for abdominal obesity. In our analyses we could only find small differences between the anthropometric measures regarding depression (PHQ-9 total score). We expected a closer relation between obesity and depression of the measures taking into account abdominal obesity compared to BMI, especially for somatic-affective symptoms of depression. After full adjustment for potential confounders in a general population between 35 to 75 years associations between the total score (PHQ-9) of depressive symptoms and different measures of obesity (BMI, WC, WHtR, WHR) were found in men and women. This finding corresponds to a recent community based meta-analysis which has found a positive relationship between obesity and depression [15]. The sex related difference regarding WHR (related to depressive symptoms in men, but not in women) remains unclear. Possibly, different body compositions as reflected in anthropometric measures might be related to sex specific affective connotations and depressive symptoms.

Differentiating the depressive symptoms in somatic-affective and cognitive-affective symptoms, we found that there was a strong positive association between somatic-affective symptoms and BMI, WC, WHtR and WHR (only in men). The relations between cognitive-affective symptoms and anthropometric measures however, ranged from none to slightly negative, and they were all lower than the relations between the somatic-affective symptoms and the anthropometric measures. Overall, this finding corresponds to the results of a recent study reporting only modest relations between cognitive-affective symptoms of depression and obesity in participants aged between 50 and 70 years [23] in a larger sample of younger obese starting at the age of 35.

The strong associations between somatic-affective symptoms of depression and obesity and especially visceral obesity may indicate an organic etiology of these symptoms of depression. It can be assumed that visceral adipose tissue plays a key role in the relationship between obesity, depression and cardiovascular disease via a higher production of pro-inflammatory cytokines. Inflammation is hypothesized to specifically drive somatic, but not cognitive-affective dimensions of depression, providing one mechanism underlying the relationship of depression and cardiovascular disease [10].

The main limitation of our study pertains to the cross-sectional data acquisition. Therefore, causal inferences are not possible. Selection bias might have occurred regarding the population with the lower severity of depressive symptoms. Therefore, our results might not be generalizable to persons with major depressive disorders. We found a relatively high rate of missing data for physical activity which might reflect problems of the SQUASH questionnaire weakening the results of our regression models. Because of the relevance of physical activity for body weight, we decided to leave this variable in the regression analyses. Results omitting physical activity as predictor (not reported in this paper) were very similar to those reported. A further limitation addresses the assessment of visceral fat only by an indirect anthropometric measure (WC). A computed tomography to identify the amount of visceral adipose tissue would have been desirable e.g. [38]. As the focus was on different measures of obesity and of depression, we did not include measures of pro-inflammatory cytokines into our analyses. As these analyses were explorative with a total of 24 comparisons (fully adjusted models), there is a risk of finding relations by chance. The strengths are a) the well characterized, representative sample of participants living in the Rhine-Main region in Germany b) the inclusion of younger participants starting at the age of 35 years and b) the relatively large sample size.

Further work on the relationship of obesity and depression should also a) include measures of visceral obesity, b) differentiate the somatic and the cognitive symptoms of depression, c) differentiate sex, d) focus on the mechanisms relating depression and abdominal obesity (e.g. genetics, pro-inflammatory cytokines) and analyze additional moderators (characteristic of specific disorders, e.g. sarcopenia in the elderly with diabetes) potentially influencing the relation between measures of obesity and depressive symptoms in a longitudinal approach and e) target interventions to prevent and reduce abdominal obesity, respectively reduce depression in obese without inducing weight gain (as e.g. by several antidepressants).

## Competing interests

All authors declare that they have no competing interest relevant to the subject matter of the manuscript that have occurred over the last two years, or that are expected in the foreseeable future.

## Authors’ contributions

JW did the first draft of the manuscript. JW, MM and MEB did the final draft of the manuscript and critically revised it for its intellectual content. IZ, AS, MB and YK substantially contributed to the analysis and interpretation of the data. PSW, MB, TM and MEB substantially contributed to the conception and the design of the study. All authors read and approved the final manuscript.

## Pre-publication history

The pre-publication history for this paper can be accessed here:

http://www.biomedcentral.com/1471-244X/13/223/prepub
